# Exploring Neurohormonal Modulation by Acetazolamide in Heart Failure

**DOI:** 10.7759/cureus.75786

**Published:** 2024-12-16

**Authors:** Midhat Asif, Fatima Malik, Abdul Salar Khan, Saadia Zainab, Muhammad Ali, Ibrahim Shah, Muhammad Ahmad Mughal, FNU Avinash, Sanjay Kirshan Kumar

**Affiliations:** 1 Medicine, Khawaja Muhammad Safdar Medical College, Sialkot, PAK; 2 Obstetrics and Gynecology, Combined Military Hospital, Sialkot, PAK; 3 Cardiology, Hayatabad Medical Complex, Peshawar, PAK; 4 Physiology, Mohi-ud-Din Islamic Medical College, Mirpur, PAK; 5 Cardiology, Rawalpindi Institute of Cardiology, Rawalpindi, PAK; 6 Cardiology, Riphah International University Railway Hospital, Rawalpindi, PAK; 7 Cardiology, Gajju Khan Medical College, Swabi, PAK; 8 Community Medicine, Wah Medical College, Wah, PAK; 9 General Medicine, Medlux Medical Center, Abu Dhabi, ARE; 10 Gastroenterolgy, Sindh Institute of Urology and Transplantation, Karachi, PAK; 11 Medicine and Allied Health, Bahria University Medical and Dental College, Karachi, PAK

**Keywords:** acetazolamide, chloride excretion, heart failure, neurohormonal modulation, renin-angiotensin-aldosterone system (raas)

## Abstract

Background

Heart failure (HF) is commonly managed by addressing water and sodium (Na) balance, with arterial circulation playing a major role in influencing renal Na and water excretion. Recently, chloride (Cl) has been recognized as an important factor in HF, associated with volume regulation and its modulation of renin-angiotensin-aldosterone system (RAAS) activity through macula densa signaling, which impacts Na retention and neurohormonal activation. Acetazolamide, a carbonic anhydrase inhibitor, can enhance decongestion in HF by increasing urinary Na and Cl excretion when added to loop diuretics, a mechanism supported by prior studies demonstrating improved urine output and decongestion.

Objective

This study investigates the neurohormonal effects of acetazolamide in acute HF, focusing on its ability to enhance decongestion, reduce neurohormonal activation (e.g., renin and aldosterone), and modulate RAAS markers.

Methods

In this prospective, single-center observational study, 80 patients with acute HF were enrolled and divided into two groups: a case group (n=40) receiving acetazolamide with standard therapy and a control group (n=40) on standard therapy alone. Patients were matched based on clinical characteristics to reduce selection bias. Baseline characteristics, neurohormonal profiles, including plasma renin activity (PRA) and aldosterone, electrolyte levels, and clinical outcomes were compared.

Results

The acetazolamide group exhibited higher urinary Cl excretion (108.9±25.3 mEq/L vs. 79.2±22.7 mEq/L; p<0.001) and reduced PRA and aldosterone levels (1.3±0.4 ng/mL/h and 88±21 pg/mL) compared to controls (1.7±0.6 ng/mL/h and 128±29 pg/mL; p=0.002 and p=0.006, respectively). These reductions in PRA and aldosterone are significant as they correlate with improved volume status and reduced neurohormonal stress, which are critical components in HF management. Improved clinical outcomes included a greater percentage of patients becoming symptom-free within 72 hours (77.5% vs. 52.5%; p=0.018) and shorter hospitalization (5.6±1.4 days vs. 7.1±1.7 days; p=0.028).

Conclusion

Acetazolamide in addition to standard therapy enhances decongestion and reduces neurohormonal activation in acute HF, suggesting its dual benefit in fluid management and neurohormonal modulation. Further research is needed to confirm these benefits, assess long-term effects, and overcome limitations, including the study's single-center observational design.

## Introduction

The majority of research on heart failure (HF) syndrome has focused on managing body fluid dynamics by controlling water and sodium (Na) balance [1]. According to a well-established theory of body fluid regulation in HF, arterial circulation is the main bodily fluid compartment influencing renal Na and water excretion [2,3]. However, chloride (Cl), despite being the anionic equivalent of Na in salt, plays a more nuanced role in HF pathogenesis that warrants further exploration. Cl was largely overlooked until 2015, when its significance in HF pathogenesis was recognized [4]. Emerging evidence now highlights Cl as a key prognostic marker, with hypochloremia in acute and chronic HF being linked to poor clinical outcomes, including rehospitalization and mortality [5-7]. Additionally, Cl is considered a key regulator of body fluid distribution [8,9]. Recent studies suggest two types of HF progression, increased vs. non-increased serum Cl concentration ([sCl−]), based on Cl's role in vascular/cellular volume regulation [10,11]. Cl also plays a central role in the activation of the renin-angiotensin-aldosterone system (RAAS) by modulating macula densa signaling, which influences renal Na retention and volume overload. Dysregulated RAAS activity contributes significantly to HF pathophysiology, making Cl a pivotal factor in managing neurohormonal pathways [12].

Current guidelines recommend intravenous loop diuretics to relieve fluid overload symptoms in patients with acute decompensated HF [7]. However, even with high-dose loop diuretics, many patients are discharged with persistent volume overload, a strong predictor of poor outcomes [13]. For example, persistent congestion is associated with higher rates of rehospitalization and mortality, underscoring the need for more effective decongestion strategies. Only 15% of patients in the Diuretic Optimization Strategies Evaluation (DOSE) trial were free of congestion after 72 hours of treatment [13]. Moreover, approximately 20% of patients in the Acute Decompensated Heart Failure National Registry (ADHERE) were discharged with weight gain, indicating unresolved fluid overload [14].

Sequential diuretic therapy has been suggested [15] as a more effective decongestive approach than loop diuretics alone, but limited information exists on optimal agents, dosing, and administration methods. When combined with loop diuretics, acetazolamide, a carbonic anhydrase inhibitor, decreases proximal tubular Na reabsorption, potentially enhancing decongestion by increasing diuretic efficacy. Prior studies on acetazolamide, including an observational study with a small sample size and a randomized trial, demonstrated increased urine Na excretion when 500 mg of intravenous acetazolamide was added to loop diuretic therapy in acute HF patients. However, these studies were limited by small cohorts and lacked detailed neurohormonal assessments [15]. Understanding how acetazolamide modulates neurohormonal pathways is critical, as RAAS and other neurohormonal systems are pivotal in both acute and long-term HF management, with significant implications for clinical outcomes such as symptom relief and hospitalization duration. This study aims to explore the neurohormonal modulation by acetazolamide in HF, providing insights into its potential to enhance decongestion and improve outcomes in HF patients.

## Materials and methods

Study design

This prospective, single-center observational study was conducted at Hayatabad Medical Complex, Peshawar, Pakistan, over a six-month period from January 2024 to June 2024. A sample size of 80 patients was calculated using the formula $\text{n}=\left(Z_{&alpha;/2}+Z_{&beta;​}\right)^{2}&sdot;\left(2.\sigma^{2}\right)/\Delta^{2}$, where σ is the standard deviation of the primary outcome, Δ is the effect size, $Z_{&alpha;/2}$=1.96 for a significance level of 0.05, and $Z_{&beta;​}$=0.84 for 80% power. The assumed effect size of 0.5 was derived from prior studies on acetazolamide's effects in HF. Patients with acute HF were allocated into the case and control groups based on clinical discretion, as randomization was not performed. This observational allocation method may introduce selection bias, which is acknowledged as a study limitation. The case group comprised patients treated with acetazolamide in addition to standard HF therapy, and the control group received standard HF therapy without acetazolamide in accordance with the European Society of Cardiology (ESC) guidelines for the diagnosis and treatment of acute and chronic HF [16,17].

Inclusion and exclusion criteria

Adult patients aged 18 years or older with acute HF were included, defined by the presence of at least two clinical signs, such as third heart sound, pulmonary crackles, leg edema, weight gain of at least 1.5 kg, or ultrasound-confirmed pleural effusion. The diagnostic criteria for acute HF also included plasma brain natriuretic peptide (BNP) levels >100 pg/mL or echocardiographic evidence of left ventricular dysfunction. Patients with cardiogenic shock, acute coronary syndrome, or severe renal impairment (defined as serum creatinine >3 mg/dL in accordance with Kidney Disease: Improving Global Outcomes (KDIGO) guidelines) were excluded. The Institutional Review Board of Hayatabad Medical Complex approved the study (reference number: 1455-3), and written informed consent was obtained from all participants.

Sample collection and parameters

Upon admission and before the initiation of decongestive therapy, patients underwent a physical examination, blood tests, and a spot urine test. Blood and urine samples were collected after a 20-minute resting period in a supine or semi-supine position. Peripheral blood tests included hemoglobin, hematocrit, blood urea nitrogen, electrolytes (Na, potassium, and Cl), and creatinine levels. Neurohormonal modulation was assessed by measuring plasma neurohormones, including noradrenaline, renin, aldosterone, and arginine vasopressin (AVP). Duarte's formula was chosen to estimate plasma volume status due to its practicality in a clinical setting, especially when direct measurements are not feasible. However, we acknowledge that this method may have limitations such as potential variability in hematocrit and hemoglobin measurements that can influence accuracy [18]. The formula used was as follows: estimated plasma volume status (dL/g)=[100−hematocrit (%)]/hemoglobin (g/dL).

Spot urine tests evaluated osmolality, creatinine, and electrolyte levels. Biomarkers such as plasma BNP were measured using chemiluminescent immunoassay. Noradrenaline and adrenaline levels were determined through high-performance liquid chromatography, while plasma renin activity (PRA) was analyzed by enzyme immunoassay. We recognize that methods such as radioimmunoassay for vasopressin may introduce variability due to its sensitivity and dependence on assay conditions. Aldosterone and AVP levels were measured using radioimmunoassay. Fractional electrolyte excretions were calculated as fractional excretion of X=(Xurine/Xserum)×(Crserum/Crurine)×100 [19,20]. Urine osmotic pressure was determined using an automatic osmotic pressure measuring apparatus (OM-6060, Arkray Inc., Kyoto, Japan) through the freezing point depression technique.

Tubular reabsorption of Cl was assessed using serum and urine Cl concentrations. Patients were classified as having low avidity (high urinary Cl levels) if the difference between serum and urinary Cl concentrations was less than zero or high avidity (low urinary Cl levels) if the difference was zero or greater, based on Martens' concept of renal Na avidity [21]. Follow-up measurements were not performed after therapy initiation or at discharge, and this has been acknowledged as a limitation in the study.

## Results

Patient characteristics

Eighty patients with acute HF were enrolled and divided into two groups: the case group (n=40), who received acetazolamide in addition to standard therapy, and the control group (n=40), who received standard therapy alone. Baseline characteristics, including age, gender distribution, and New York Heart Association (NYHA) classification, were comparable between the two groups, with no statistically significant differences. The median BNP levels were also similar between the groups (810 (620-1210) pg/mL in the case group vs. 840 (660-1290) pg/mL in the control group; p=0.83) (Table 1).

**Table 1:** Baseline characteristics of the study population *: independent t-test; #: Fisher's exact test; †: Mann-Whitney U test; NYHA: New York Heart Association; HF: heart failure; BNP: brain natriuretic peptide; ACE: angiotensin-converting enzyme; ARBs: angiotensin II receptor blockers Values are significant at p≤0.05. The BNP distribution was assessed for skewness and normality using the Shapiro-Wilk test, which indicated a non-normal distribution (p<0.05). Hence, the Mann-Whitney U test was used for comparison. The high prevalence of hypertension (57.5%), ischemic heart disease (22.5-27.5%), and valvular disease (12.5-20%) align with typical etiologies of acute HF in this region, improving generalizability to similar populations. Additional data on diabetes, chronic kidney disease, and standard baseline treatments (ACE inhibitors/ARBs, beta-blockers, and diuretics) were included, as these could influence outcomes and provide a more comprehensive understanding of the study population.

Characteristic	Case group (with acetazolamide) (n=40)	Control group (without acetazolamide) (n=40)	P-value
Age, years (mean±SD)	70.3±10.2	69.5±9.9	0.64*
Male, n (%)	23 (57.5%)	21 (52.5%)	0.68#
NYHA class III/IV, n (%)	36 (90%)	33 (82.5%)	0.75#
Primary cause of HF			
Hypertension, n (%)	23 (57.5%)	21 (52.5%)	0.65#
Ischemic heart disease, n (%)	9 (22.5%)	11 (27.5%)	0.63#
Valvular disease, n (%)	5 (12.5%)	8 (20%)	0.77#
Atrial fibrillation, n (%)	19 (47.5%)	17 (42.5%)	0.72#
BNP (pg/mL), median (IQR)	810 (620-1210)	840 (660-1290)	0.83†
Other baseline comorbidities			
Diabetes, n (%)	18 (45%)	16 (40%)	0.69#
Chronic kidney disease, n (%)	7 (17.5%)	9 (22.5%)	0.78#
Baseline treatments			
ACE inhibitors/ARBs, n (%)	29 (72.5%)	27 (67.5%)	0.74#
Beta-blockers, n (%)	26 (65%)	25 (62.5%)	0.83#
Diuretics, n (%)	36 (90%)	34 (85%)	0.76#

Neurohormonal profile

The neurohormonal analysis revealed significant differences in PRA and aldosterone levels between the groups. The case group showed lower PRA (1.3±0.4 ng/mL/h) compared to the control group (1.7±0.6 ng/mL/h; p=0.002), indicating reduced neurohormonal activation. Similarly, aldosterone levels were significantly lower in the case group (88±21 pg/mL) compared to the control group (128±29 pg/mL; p=0.006). There were no significant differences between groups in noradrenaline or AVP levels (p=0.12 and p=0.28, respectively) (Table 2). The absence of significant differences in noradrenaline and AVP levels may reflect limitations such as sample size, which could reduce statistical power, or potential variability in the sensitivity of measurement techniques used.

The observed reductions in PRA and aldosterone levels in the case group suggest a potential attenuation of neurohormonal activation, which is a key driver of HF progression. Lower levels of these parameters may indicate improved hemodynamic stability and reduced cardiac workload, aligning with the therapeutic goals in HF management.

**Table 2:** Neurohormonal profile of the study groups PRA: plasma renin activity; AVP: arginine vasopressin Independent t-test is applied. Values are mean±SD or median and significant at p≤0.05.

Neurohormonal parameter	Case group (with acetazolamide) (n=40)	Control group (without acetazolamide) (n=40)	P-value
Noradrenaline (pg/mL)	425±81	455±91	0.12
PRA (ng/mL/h)	1.3±0.4	1.7±0.6	0.002
Aldosterone (pg/mL)	88±21	128±29	0.006
AVP (pg/mL)	2.7±1.1	3.0±1.3	0.28

Electrolyte and volume status

Analysis of electrolyte and volume status indicated that patients in the case group had significantly higher urinary Cl excretion (108.9±25.3 mEq/L) than those in the control group (79.2±22.7 mEq/L; p<0.001). Additionally, the fractional excretion of Cl was higher in the case group (4.3±1.4%) compared to the control group (2.3±1.1%; p<0.001), reflecting improved Cl clearance. The serum-to-urine Cl difference also significantly favored the acetazolamide-treated group, with a negative value in the case group (-6.3±4.7 mEq/L) versus a positive difference in the control group (8.6±5.1 mEq/L; p<0.001) (Table 3). Clinically, a higher fractional excretion of Cl in the case group indicates improved renal Cl clearance, which is associated with more effective decongestion and reduced systemic congestion. This metric provides a dynamic reflection of diuretic efficacy, complementing traditional volume status indicators.

**Table 3:** Electrolyte and volume status of the study groups Na: sodium; K: potassium; Cl: chlorine Independent t-test is applied. Values are mean±SD and significant at p≤0.05.

Parameter	Case group (with acetazolamide) (n=40)	Control group (without acetazolamide) (n=40)	P-value
Serum Na (mEq/L)	139.2±4.1	138.6±4.4	0.38
Serum K (mEq/L)	4.4±0.4	4.3±0.5	0.53
Serum Cl (mEq/L)	103.8±5.1	104.1±5.0	0.63
Urinary Cl (mEq/L)	108.9±25.3	79.2±22.7	<0.001
Fractional excretion of Cl (%)	4.3±1.4	2.3±1.1	<0.001
Serum Cl-urinary Cl (mEq/L)	-6.3±4.7	8.6±5.1	<0.001

Clinical outcomes

Assessed clinical outcomes included symptom relief, weight reduction, duration of hospitalization, and percentage reduction in BNP levels. A higher proportion of patients in the case group were symptom-free within 72 hours (31 patients, 77.5%) compared to the control group (21 patients, 52.5%), with a statistically significant difference (p=0.018). Symptom-free status was defined as the absence of dyspnea at rest, a stable NYHA functional class improvement by one level, and the discontinuation of intravenous diuretics for >24 hours. Weight reduction was also greater in the case group (1.9±0.7 kg) than in the control group (1.3±0.6 kg; p=0.014), likely reflecting improved decongestion and fluid balance, which are key goals in acute HF management. The observed weight reduction correlated with symptom relief and enhanced volume status, underscoring its clinical relevance.

The case group had a shorter hospitalization duration (5.6±1.4 days) compared to the control group (7.1±1.7 days; p=0.028). Although this difference is statistically significant, it reflects trends rather than definitive conclusions due to the comparative nature of this analysis. BNP reduction was also greater in the acetazolamide group (34±16%) compared to the control group (26±11%; p=0.045). While statistically significant, the clinical implications of this reduction must be interpreted cautiously. Reductions in BNP above 30% are often associated with improved HF outcomes, although thresholds for clinical improvement vary across studies and patient populations (Table 4).

**Table 4:** Clinical outcomes #: Fisher's exact test for categorical variables; *: independent t-tests for continuous variables Values are presented as mean±SD or n (%) and significant at p≤0.05.

Outcome	Case group (with acetazolamide) (n=40)	Control group (without acetazolamide) (n=40)	P-value
Symptom-free after 72 hours	31 (77.5%)	21 (52.5%)	0.018#
Weight reduction (kg)	1.9±0.7	1.3±0.6	0.014*
Hospitalization duration (days)	5.6±1.4	7.1±1.7	0.028*
BNP reduction (%)	34±16	26±11	0.045*

## Discussion

This study demonstrates that acetazolamide, when added to standard therapy in acute HF, is associated with significantly improved decongestion, enhanced urinary Cl excretion, and reduced neurohormonal activation. Acetazolamide, a carbonic anhydrase inhibitor, acts primarily on the proximal tubule, inhibiting Na and bicarbonate reabsorption. This mechanism is particularly valuable in acute HF, where achieving effective decongestion often faces challenges due to diuretic resistance or the limited efficacy of loop diuretics in targeting proximal Na and Cl reabsorption. By enhancing Cl excretion, acetazolamide complements the action of loop diuretics and adds to the therapeutic armamentarium for managing fluid overload in HF.

Patients in the acetazolamide group showed markedly higher urinary Cl excretion compared to the control group and a higher fractional excretion of Cl. This increase in Cl excretion indicates an improvement in fluid clearance and suggests that acetazolamide reduces renal avidity for Cl by inhibiting carbonic anhydrase in the proximal tubule, enhancing the distal delivery of Na and Cl for excretion. Improved Cl excretion has been associated with better volume management outcomes, such as weight reduction and symptom relief, further highlighting acetazolamide's role in decongestion strategies.

This study primarily focuses on the renal and systemic effects of SGLT2 inhibitors, particularly their interaction with renal tubular Na, water, and Cl homeostasis in the context of HF. While the study's primary focus is on SGLT2 inhibitors rather than acetazolamide, its findings underscore the significance of proximal tubular function and Cl handling in managing HF. The review synthesizes evidence demonstrating the limited acute diuresis and natriuresis associated with SGLT2 inhibitors, owing to rapid renal compensation. However, persistent effects on proximal Na and glucose reabsorption suggest a durable improvement in the internal set point for volume homeostasis [22].

Applying this analogy to acetazolamide, our study aligns with the concept that proximal tubular modulation (in our case, through carbonic anhydrase inhibition) leads to sustained improvements in fluid clearance. Unlike SGLT2 inhibitors, acetazolamide enhances Cl excretion by inhibiting reabsorption in the proximal tubule, as demonstrated by significantly higher urinary Cl levels and fractional excretion of Cl in our acetazolamide group.

Neurohormonal modulation was also significant in our study. PRA and aldosterone levels were significantly lower in the acetazolamide group than in the control group (1.7±0.6 ng/mL/h and 128±29 pg/mL; p=0.002 and p=0.006, respectively). These reductions in PRA and aldosterone levels suggest that acetazolamide indirectly dampens RAAS activity, possibly due to improved volume status and reduced neurohormonal stress on the heart. Lower RAAS activity is associated with favorable long-term HF outcomes, such as reduced hospitalization rates and mortality, as it mitigates fluid retention and systemic congestion. However, the lack of significant changes in noradrenaline (425±81 pg/mL vs. 455±91 pg/mL; p=0.12) or AVP levels (2.7±1.1 pg/mL vs. 3.0±1.3 pg/mL; p=0.28) could reflect different regulatory pathways or the limitations of the acute response to volume changes. Studies have shown that RAAS activation in HF is closely linked to fluid retention and reducing RAAS markers is associated with improved outcomes [23]. No significant differences were observed in noradrenaline (425±81 pg/mL vs. 455±91 pg/mL; p=0.12) or AVP levels (2.7±1.1 pg/mL vs. 3.0±1.3 pg/mL; p=0.28) between groups [24]. However, the specific reduction in PRA and aldosterone supports the role of acetazolamide in modulating RAAS without directly affecting sympathetic or vasopressinergic systems [25].

Clinical outcomes further demonstrated the benefits of acetazolamide. A significantly higher proportion of patients in the acetazolamide group were symptom-free at 72 hours (77.5% vs. 52.5%; p=0.018), and the acetazolamide group experienced greater weight reduction (1.9±0.7 kg vs. 1.3±0.6 kg; p=0.014). These improvements align with patient-centric goals, such as enhanced quality of life and functional status, and indicate more effective decongestion [26]. The shorter hospitalization duration observed in the acetazolamide group (5.6±1.4 days vs. 7.1±1.7 days; p=0.028) highlights its potential to reduce healthcare costs and optimize resource utilization. This aligns with findings from the ADVOR trial, which reported faster and more complete decongestion in HF patients receiving acetazolamide. Notably, the baseline characteristics and acetazolamide dosing regimens in the Acetazolamide in Decompensated Heart Failure with Volume Overload (ADVOR) trial were similar to those in this study, strengthening the comparability of results.

Limitations

This study has limitations. As a single-center, prospective study, our findings may be influenced by selection bias and may not be generalizable to other populations. Additionally, the lack of long-term follow-up restricts our understanding of acetazolamide's sustained effects on HF outcomes. The absence of a placebo control, low sample size, and potential variability in patient adherence to therapy are additional concerns. Furthermore, reliance on surrogate markers like PRA or urinary Cl limits the ability to directly assess mechanistic pathways. Differences in the studied population, such as age, comorbidities, or HF severity, might also affect the generalizability of findings to other HF cohorts.

Future perspectives

Future studies should explore acetazolamide's long-term impact on HF progression and assess its potential role in reducing hospital readmissions and mortality. Specific outcomes of interest, such as quality of life, functional status, and renal outcomes, warrant further investigation. Additionally, exploring acetazolamide's effects in HF subpopulations, such as those with preserved ejection fraction (HFpEF), diuretic resistance, or renal impairment, would help expand its applicability. Comparisons with other adjunctive therapies and evaluations of its efficacy in combination with emerging HF treatments are also needed to refine its role in HF management.

## Conclusions

Acetazolamide added to standard therapy significantly enhances decongestion, increases urinary Cl excretion, and reduces neurohormonal activation in acute HF patients. These findings suggest that acetazolamide could refine patient selection criteria for adjunctive therapy in HF, especially for those resistant to standard diuretics. Although the precise mechanistic pathways require further elucidation, our results highlight acetazolamide's clinical utility in addressing volume overload in acute HF. Larger, multicenter trials are necessary to confirm these findings, evaluate its long-term benefits, and establish its place in HF treatment guidelines.
